# Environmental and Animal Characteristics as Factors Associated with American Cutaneous Leishmaniasis in Rural Locations with Presence of Dogs, Brazil

**DOI:** 10.1371/journal.pone.0047050

**Published:** 2012-11-05

**Authors:** Norberto Assis Membrive, Gesse Rodrigues, Kezia Peres Gualda, Marcos Vinícius Zandonadi Bernal, Diego Molina Oliveira, Maria Valdrinez Campana Lonardoni, Ueslei Teodoro, Jorge Juarez Vieira Teixeira, Thaís Gomes Verzignassi Silveira

**Affiliations:** 1 Programa de Pós-Graduação em Ciências da Saúde, Universidade Estadual de Maringá, Maringá, Paraná, Brazil; 2 Laboratório de Entomologia Médica de Arapongas, Secretaria Municipal de Saúde, Arapongas, Paraná, Brazil; 3 Curso de Graduação em Farmácia, Universidade Estadual de Maringá, Maringá, Paraná, Brazil; 4 Departamento de Análises Clínicas e Biomedicina, Universidade Estadual de Maringá, Maringá, Paraná, Brazil; Centro de Pesquisa Rene Rachou/Fundação Oswaldo Cruz (Fiocruz-Minas), Brazil

## Abstract

The aim of the study was to investigate the importance of dogs, other domesticated animals and environmental characteristics as risk factors in the epidemiology of American cutaneous leishmaniasis (ACL). A retrospective survey of cases of human ACL in the last ten years and visits to homes in rural locations were carried out in the municipality of Arapongas (southern Brazil) from 2008 to 2010. ACL in humans was significantly associated with a distance of up to 25 meters from the residence to a forest area (OR 5.08; 95% CI: 1.35–21.04), undergrowth area (OR 6.80; 95% CI: 1.69–45.33) and stream (OR 5.87; 95% CI: 1.15–24.59); banana plants near the residence (OR 5.98; 95% CI: 1.49–39.84), absence of ceiling below the roof in the residence (OR 7.30; 95% CI: 1.26–158.1), the dumping of trash in the forest area (OR 26.33; 95% CI: 7.32–93.46) and presence of ACL in dogs in the surrounding area (OR 4.39; 95% CI: 1.37–13.45). In dogs, ACL was associated with a distance of 25 to 50 meters and 51 to 100 meters, respectively, from the residence to a forest area (OR 2.59; 95% CI: 1.08–5.98; OR 3.29; 95% CI: 1.64–6.62), the presence of a stream up to 25 m from the residence (OR 6.23; 95% CI: 2.34–16.54) and banana plants near the residence (OR 0.45; 95% CI: 0.25–0.80). In the locations studied in the municipality of Arapongas (Brazil), the results reveal that canine infection increases the risk of human infection by ACL and the characteristics surrounding the residence increase the risk of infection in both humans and dogs. Thus, integrated environmental management could be a useful measure to avoid contact between humans and phlebotomines.

## Introduction

Leishmaniasis threatens about 350 million people in 88 countries around the world, 72 of which are developing countries. About 12 million people are believed to be currently infected, with one to two million estimated new cases occurring every year [1]. The disease may appear in its cutaneous**,** mucocutaneous or visceral form, with a wide range of clinical symptoms. Cutaneous leishmaniasis is the most common. Ninety percent of cases of cutaneous leishmaniasis occur in Afghanistan, Brazil, Iran, Peru, Saudi Arabia and Syria and 90% of cases of mucocutaneous leishmaniasis occur in Bolivia, Brazil and Peru [1].

American cutaneous leishmaniasis (ACL) is found in all states of Brazil and its prevalence has increased considerably. ACL is endemic in the state of a Paraná (southern Brazil), with records in 289 of the 399 municipalities [2]–[4], accounting for 99.6% of the 15,443 cases in the southern region of the country between 1980 and 2006 [5]. The occurrence of ACL in humans and dogs in different municipalities in the state of Paraná underscores the need for epidemiological studies on this disease, especially in locations with environmental characteristics that favor its transmission.

The scarcity or lack of knowledge in the literature on probable factors associated with ACL demonstrates the importance of complementary studies. Few investigations have addressed risk factors [6], [7], such as the distance of residences from forest area, and fewer still have addressed bodies of water, domesticated animals, vegetation and housing conditions as risk factors for the disease [8], [9]. In the state of Paraná, studies have reported ACL infection in dogs [10]–[15], equines [16] and wild animals [17].

The aim of the present study was to investigate the importance of dogs and other domesticated animals as well as environmental characteristics as factors associated with ACL in rural locations in a municipality in the northern portion of the state of Paraná, Brazil.

## Methods

### Study Area

The municipality of Arapongas covers an area of approximately 38,000 hectares and is located in the northern of the state of Paraná, Brazil (23°24′211′′ S and 51°26′448′′ W) at 816.36 meters above sea level. The city has a population of 104,161 inhabitants (102,543 in urban areas and 1,618 in rural areas) [18]. The climate is subtropical (Köppen classification: CFa), with temperatures ranging from 32°C to 8°C. The topology consists of low, relatively non-desiccated plateaus. The soil is dark red latosol and red nitosol, with strips of well-drained sandy soil derived from the basalt. Most of the area has distroferric soil with a low degree of natural fertility. Only 2008 hectares of the original seasonal semi-deciduous forest remain as residual forest or riparian forest. Pastures account for 3500 hectares and most of the area (22,400 hectares) has been occupied by soybean, corn, wheat, coffee, oats and orange crops.

### Ethical Considerations

The present study received approval from the Human Research Ethics Committee (report 138/2007 on 18/May/2007) of the State University of Maringá. All participants were informed as to the importance and objectives of the study and were ensured both anonymity and confidentiality. Those who agreed to participate signed a statement of informed consent.

All procedures involving dogs followed the Ethical Principles of Animal Experimentation established by the Brazilian College of Animal Experimentation (COBEA) and were performed according to protocols approved by the Committee for Ethical Conduct in Animal Experimentation of the State University of Maringá (report 015/20076 on 03/Apr/2007). All owners of the dogs were informed as to the importance and objectives of the study and those who agreed to have their animals involved provided a statement of informed consent.

### Study Design and Procedures

A retrospective epidemiological survey was carried out of cases of human ACL in the last ten years registered at the Arapongas Municipal Secretary of Health. The patient notification files were analyzed and data on gender, age, profession, number and site of lesions, time and likely location of infection, diagnosis and treatment were recorded. Only cases classified as autochthonous to the municipality were included.

Between June 2008 and December 2010, all residences in rural areas of the municipality were visited for the localization of human cases of ACL that occurred in the last ten years and determination of environmental and animal characteristics. At each home, an epidemiological chart was filled out addressing the following data: number of residents in the home, type of crop, distance from residence to forest area, presence of undergrowth area, streams and shelters for animals, species and quantities of domesticated animals and presence of wild animals. All residents were given reading material on ACL, containing basic notions regarding the disease, transmission, symptoms, prophylaxis and treatment.

A total of 469 rural locations were visited. In the majority (99%) of the localities there was only one residence. There were included in the study 386 locations where had dogs, with a total of 1,392 persons and 1,103 dogs residing. The dogs were examined for general health state and the presence of lesions suggestive of ACL and registered on individual charts containing the following clinical-epidemiological information: name of animal, registry number, name of owner, address and clinical history. Dogs with ulcerated skin lesions on any part of the body and nodular lesions in areas with sparse hair were suspected of having ACL.

**Figure 1:**
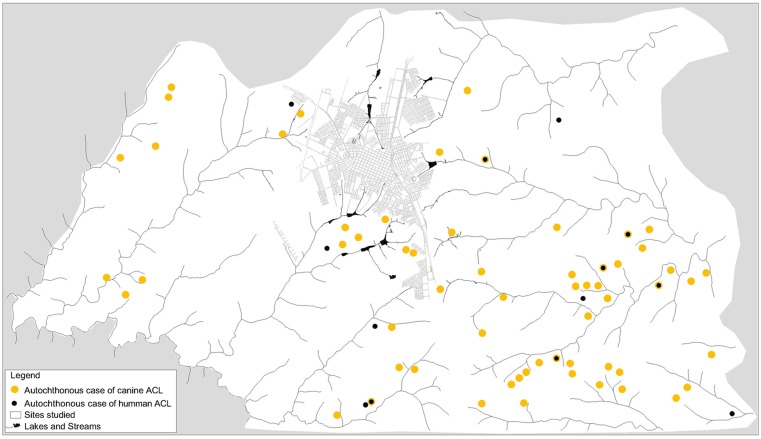
Geo-referencing of rural locations studied in the municipality of Arapongas, State of Paraná, Brazil.

The locations studied were mapped (Figure 1) using the Global Positioning System. The ArcGis 9 program (ArcReader®, version 9.3.1) was used for geo-referencing, the basis of which was the map of the municipality of Arapongas (2001).

### Collection of Biological Samples from Dogs

A blood sample (approximately 5 mL) was collected from the brachial vein of each dog. The serum was stored at −20°C until use.

Biopsies were performed on the animals with ulcerated skin lesions suggestive of ACL for the collection of biological material from the lesions. Following asepsis and an injection of 1% xylociane, the biopsy was performed with incisions measuring 2 to 5 mm along the edges of the lesions. The fragments were used for direct study, isolation of the parasite and polymerase chain reaction (PCR). Fragments for PCR analysis were kept in STE buffer (NaCl 0.1 M; TRIS 10 mM, pH 8.0; Na_2_EDTA.2H_2_O 1 mM, pH 8.0) and stored at −18°C until DNA extraction.

### Laboratory Diagnosis of ACL in Dogs

For the direct study of the parasite, fragments were smeared on slides, stained with the Giemsa method and examined under a common optical microscope.

For the isolation of the parasite, fragments were incubated in saline solution containing penicillin G potassium (Sigma) (25,000 UI/mL) and streptomycin (Sigma) (2 µg/mL) at 4°C for 24 hours. The fragments were then sectioned and cultured in biphasic NNN (Novy, MacNeal and Nicole)/Schneider’s Insect Medium (Sigma), containing 10% inactive fetal bovine serum. The cultures were incubated at 27°C for two weeks and analyzed weekly for parasite growth. Other fragments were macerated in saline solution containing penicillin G potassium (Sigma) (25,000 UI/mL) and streptomycin (Sigma) (2 µg/mL) and 0.1 ml of the macerated suspension was inoculated in the footpad of hamsters (*Mesocricetus auratus*).

The indirect immunofluorescence reaction for leishmaniasis was performed using promastigotes of *L. braziliensis* and anti-dog immunoglobulin G conjugated to fluorescein (Sigma), considering counts ≥40 to be significant [12]. The Imunocruzi antigen (Biolab, Rio de Janeiro, Brazil) and anti-dog immunoglobulin G conjugated to fluorescein (Sigma) were used for the study of anti-*Trypanosoma cruzi* antibodies.

DNA from the lesion samples was extracted using the Puregene® kit (Gentra, USA). For the PCR, the primers MP3H (5′-GAA CGG GGT TTC TGT ATG C-3′) and MP1L (5′-TAC TCC CCG ACA TGC CTC TG-3′) were used, which amplify a 70-bp fragment of the minicircle of the kDNA of parasites of the subgenus *Leishmania (Viannia)* [19] and the reaction was performed following the method described by Massunari et al. [15].

### Statistical Analyses

The data were entered into the EpiData® 3.5.1 program and the statistical analysis was performed using the Openepi® 2.3 program, with the level of significance set at 5% (p<0.05). Pearson’s chi-squared test was used to determine associations between cases of ACL and environmental characteristics.

## Results

The present study involved 386 rural locations with dogs in the municipality of Arapongas, Brazil. A total of 1103 dogs were examined, 74.9% of which were males and 25.1% were females. Cases of autochthonous human ACL, that occurs from January 1999 to December 2008, were recorded in 14 of these rural locations.

Other domesticated animals were found in the 386 locations: chickens (301 locations; 77.9%), pigs (237 locations; 61.3%), cats (224 locations; 58.0%), equines (139 locations; 36.0%), sheep (16 locations; 4.1%), goats (9 locations; 2.8%) and others (rabbits, pheasants, turkeys and geese; 20 locations; 5.1%). Residents in 286 locations (74.0%) reported the presence of rodents in or surrounding the home. The following environmental characteristics were observed in the locations: presence of residual or riparian forests (340 locations; 88.1%), undergrowth area (323 locations; 83.1%), coffee plantations (121 locations; 31.3%), banana plants (332 locations; 86.0%), streams (338 locations; 87.5%), hen sheds (274 locations; 70.9%) and animal shelters (244 locations; 63.0%). The distance of these ecotopes from the residences ranged from 2 to 1000 meters.

Among the 1103 dogs examined, 74 (6.7%) had significant anti-*Leishmania* antibody titers. All 74 samples were tested for reactivity for *T. cruzi*, for which only five (6.7%) had counts greater than those found for *Leishmania*. Dermatological lesions suggestive of ACL were found in 48 dogs and located on the ear, scrotum, snout and mouth. Most were single ulcerated lesions. Among the 30 dogs submitted to biopsies, 25 (83.3%) exhibited amastigotes of *Leishmania* sp. in the direct parasitological exam. Among the 30 hamsters inoculated with macerated material from the biopsies, nine (30.0%) developed lesions from which the isolation of the parasite was possible. Among the 30 biopsy samples cultured in the culture medium, promastigotes of *Leishmania* sp. were isolated from one sample (3.3%). PCR analysis was positive in 25 dogs (83.3%). Among the 1055 dogs without lesions, the indirect immunofluorescence reaction was positive in 47. Among the 1,103 dogs, 91(8.3%) had at least one positive laboratory exam. Table 1 displays the results of the laboratory diagnosis.

**Table 1:** Results of laboratory exams used for diagnosis of cutaneous leishmaniasis in canine population in rural locations of the municipality of Arapongas, State of Paraná, Brazil.

Dogs	Parasite search(positive/total)	PCR in lesion(positive/total)	IIF(positive/total)	At least one positive test
With lesion (n = 48)	25/30	25/30	27/48	44/48
Without lesion (n = 1055)	–	–	47/1055	47/1055
Total	25/30	25/30	74/1103	91/1103

PCR: polymerase chain reaction.

IIF: indirect immunofluorescence reaction.

The results of the laboratory exams of the animal and autochthonous human cases in these locations allowed the identification of 68 locations with cases of ACL. Human cases alone occurred in eight locations, human and canine cases together occurred in six locations and canine cases alone occurred in 54 locations.

The analysis of associations between environmental factors and the locations in which cases of ACL occurred revealed that the presence of a forest up to 25 meters from the residence represented a 5.08-fold greater risk (95% CI: 1.35–21.04) for human cases in comparison to locations in which this distance was longer. For canines, a distance of 25 to 50 meters represented a 2.9-fold greater risk (95% CI: 1.08–5.98) and a distance of 51 to 100 meters represented a 3.29-fold greater risk (95% CI: 1.64–6.62) in comparison to other distances. A banana plants found up to 10 meters from the residence represented a 5.98-fold greater risk (95% CI: 1.49–39.84) for human cases and a 0.45-fold risk for dogs (95% CI: 0.25–0.80) in relation to greater distances. An undergrowth area at a distance of up to 25 meters from the residence represented a 6.80-fold greater risk for human cases (95% CI: 1.69–45.33) in comparison to longer distances. A stream found up to 25 meters from the residence represented a 5.87-fold greater risk for human cases (95%: 1.15–24.59) and a 6.23-fold greater risk (95% IC: 2.34–16.54) for canine cases (Table 2).

**Table 2:** Environmental characteristics as risk factors for human and canine cutaneous leishmaniasis in rural locations of the municipality of Arapongas, State of Paraná, Brazil.

Characteristics	Cases of human ACL in locations				Cases of canine ACL in locations				
	Presence	Absence	OR (95% CI)	p-value*	Presence	Absence	OR (95% IC)	p-value*	
Distance between the residence and forest (meters)									
>100	4	198	1		20	182	1		
51–100	1	74	0.67 (0.03–5.43)	0.79	20	55	3.29 (1.64–6.62)	<0.001*	
25–50	3	42	3.51 (0.63–17.59)	0.14	10	35	2.59 (1.08–5.98)	0.03*	
1–25	6	58	5.08 (1.35–21.04)	<0.02*	10	54	1.68 (0.72–3.79)	0.22	
Distance between the residence and undergrowth (meters)									
>25	2	198	1		33	167	1		
1–25	12	174	6.80 (1.69–45.33)	<0.01*	27	159	0.86 (0.49–1.50)	0.59	
Distance between the residence and stream (meters)									
>100	7	236	1		35	208	1		
51–100	1	75	0.45 (0.02–2.98)	0.50	7	69	0.64 (0.25–1.47)	0.31	
26–50	3	44	2.29 (0.47–9.07)	0.27	8	39	1.29 (0.52–2.94)	0.55	
1–25	3	17	5.87 (1.15–24.59)	0.04*	10	10	6.23 (2.34–16.54)	<0.001*	
Distance between the residence and banana plants (meters)									
>10	2	186	1		39	149	1		
1–10	12	186	5.98 (1.49–39.84)	<0.01*	21	177	0.45 (0.25–0.80)	<0.01*	
Distance between the residence and pigsty (meters)									
>10	13	273	1		39	247	1		
≤10	1	99	0.21 (0.01–1.24)	0.09	21	79	1.68 (0.92–3.02)	0.09	
Distance between the residence and hen shed (meters)									
>10	08	230	1		32	206			
≤10	06	142	1.21 (0.39–3.65)	0.72	28	120	1.50 (0.86–2.62)	0.16	
Ceiling in the residence									
Absence	13	238	7.30 (1.26–158.1)	0.02*	16	119	1		
Presence	1	134	1		44	207	1.58 (0.86–2.99)	0.14	
Trash dumped in the forest									
No	8	362	1		56	314	1		
Yes	6	10	26.33 (7.32–93.46)	<0.001*	4	12	1.86 (0.50–5.79)	0.31	
Animals in and around the home									
Absence	2	90	1		12	80	1		
Rodents	10	152	2.95 (0.70–20.18)	0.16	20	142	0.94 (0.44–2.08)	0.86	
Opossums	0	9	–	0.83	0	9	-	0.81	
Rodents and opossums	2	121	0.74 (0.08–7.27)	0.78	28	95	1.96 (0.94–4.23)	0.07	
Rodents and/or opossums	12	282	1.91 (0.47–12.80)	0.43	48	246	1.30 (0.67–2.66)	0.46	
Cases of canine ACL in location									
Absence	8	318	1						
Presence	6	54	4.39 (1.37–13.45)	0.01*					
Number of dogs per location									
1					4	104	1		
2–5					41	202	5.26 (1.98–17.66)	<0.001*	
6–9					10	18	14.00 (4.05–56.51)	<0.001*	
≥10					5	2	57.19 (8.81–536.71)	<0.001*	

OR: Odds Ratio, CI: Confidence interval, * Significant association (p<0.05).

Regarding the characteristics of the area surrounding the residence, a home without a ceiling below the ceramic tiled roof represented a 7.30-fold greater risk (95%: 1.26–158.1) for human cases in comparison to a home with a ceiling (p = 0.02). Locations where household trash was dumped in the forest area represented a 26.33-fold greater risk (95% CI: 7.32–93.46) for human cases in comparison to locations where trash was burned, buried or collected by the public sanitation service. The presence of rodents and/or opossums in or around the home not represented a risk for infection. The presence of canine cases represented a 4.39-fold greater risk (95% CI: 1.37–13.45) for human cases. The risk for infection of dogs increased as increasing the number of dogs by location, ranging from 5.26 to 57.19 (Table 2).

## Discussion

The transmission cycle of *Leishmania* exhibits characteristics that are particular to each endemic area, which does not always allow the extrapolation of data from one region to another. On the other hand, many endemic areas with similar environmental characteristics share details such as the same species of parasite, wild mammals (reservoirs) and phlebotomines (vectors) [20].

The domesticated animals found in the 386 locations included dogs, chickens, pigs, cattle, equines, sheep, goat and cats, which may serve as a source of blood for female phlebotomines. Chickens are refractory to infection by *Leishmania* [21]. There are no reports of the isolation of *Leishmania* in cats in southern Brazil, but *Leishmania (Viannia)* DNA has been detected in equines [16]. Domesticated animals in the area surrounding residences attract phlebotomines [22] and the presence of these animals has been reported in a number of locations where cases of ACL have occurred [23], [24]. Similar situation have also been observed in other regions of Brazil [25]–[27]. In previous work, in rural areas of the municipality of Arapongas, sandflies were collected with predominance of *Lutzomyia whitmani* [28].

In 10 locations where cases of human ACL occurred and 40 locations where canine cases occurred, the residences were within 100 meters from forest areas. A distance of up to 25 meters between the residence and forest was a risk factor for human infection, whereas a distance of 25 to 100 meters was a risk factor for canine cases. The proximity between the residence and forest within the flight range of phlebotomines makes humans and dogs available sources of food for females at night. Long-distance flights are believed to be due to a lack of food and adequate sites for oviposition [29]. In environments in which such conditions are adequate, there is a lesser tendency for phlebotomines to leave these ecotopes, which increases the risk of infection in humans and dogs.

Undergrowth area found within 25 meters from a residence increased the risk of infection in humans in comparison to longer distances. Despite undergoing constant modifications by humans, undergrowth may serve as habitats for wild animals. Barros et al. [30] found a high frequency of phlebotomines in undergrowth, especially *L. whitmani,* throughout the entire year, regardless of the season. Undergrowths may be merely resting grounds for phlebotomines. However, as these areas form an environment with moist soil and organic matter, undergrowth may serve as nurseries for these insects in the area surrounding residences [30].

A residence located up to 25 meters from a stream may favor both human and canine infection. Streams offer a biologically ideal habitat for phlebotomines, providing essential conditions for development, such as moisture, oxygen and decomposing organic matter [29]. Thus, the proximity of a stream leads to an increase in the frequency of phlebotomines and enables the occurrence of new cases of ACL in and around the home.

The presence of banana plants within 10 meters of residences was a risk factor for both human and canine cases. Pedrosa and Ximenes [8] report the frequent presence of banana plants in areas of ACL transmission in the state of Alagoas (northeastern Brazil). According to Aguiar and Medeiros [31], the species *Lutzomyia intermedia* and *L. migonei,* which are frequent in the state of Paraná [32], are associated with banana crops. Bananas have constituted a subsistence crop in the northern portion of Paraná since the time of colonization. Due to the accentuated alterations caused by human actions, banana plants may be replaced with natural habitats that can serve as phlebotomine nurseries. However, this can be avoided with measures such as the removal of organic matter (stems and leaves) from the plantation following the harvesting of the fruit, thereby avoiding the occurrence of moist soil in shaded areas. One case of ACL occurred in a location where the residence was 486 meters from forest area and five meters from a banana plants, in which the patient was unable to leave the residence due to physical limitations. The residents in this location did not remove the stems and leaves following the harvesting of the fruit and the interior of the plantations had *L. whitmani* [28], which is involved in the transmission of ACL in the state of Paraná.

The presence of animal shelters and hen sheds 10 meters from the residence did not prove to be associated with cases of ACL in humans or dogs. However, the high frequency of phlebotomines reported in shelters for domesticated animals demonstrates that this type of construction attracts these insects [22], [32].

Among the total number of locations studied, 65.0% had homes without a ceiling below the ceramic tiled roof. The absence of a ceiling represented a 7.30-fold greater risk of human ACL. The vector can easily enter these homes at night, attracted by the light in the interior of the residence, as all locations studied have electricity [22]. Thus, a ceiling can both improve the quality of the habitation and contribute toward reducing the frequency of ACL.

In 16 (4.1%) of the locations, household trash was dumped on the margins or interior of forest. While this is a small percentage in relation to the total number of locations (386), this practice led to a 26.33-fold greater chance of humans contracting ACL in comparison to locations in which the residents habitually burned, buried or recycled trash. Among the locations in which trash was dumped in the forest, one small farm had five cases and one site had 11 cases of human ACL. Discarding trash in the forest also contributes toward an increase in the rodent population in the same environment in which the procreation of phlebotomines takes place. The maintenance of the *Leishmania* transmission cycle may be due to natural reservoirs in the surrounding forest, such as some rodent species [5], [33], [34]. The correct disposal of household trash can serve as a protection measure by reducing the rodent population.

In 73.8% of the locations studied, the residents reported the presence of a large number of rodents and/or opossums in and around the home, but this finding was not associated with risk of infection. Despite the lack of a significant association, it should be mentioned that natural infection by *Leishmania* (*V.) braziliensis* has been detected in a rodent of the genus *Nectomys* [35] in one of the locations studied in the present investigation. In the same region, opossums (*Didelphis albiventris*) with anti-*Leishmania* antibodies have been detected [17]. In the northern region of Brazil, it was caught opossums (*Didelphis marsupialis*) with lesions and hemoflagellates that could not be characterized and suggest that opossums may be an important link in the transmission of disease as a secondary reservoir of *Leishmania (V.) guyanensis* [36]. However, in another study carried out in the same region, the presence of opossums around the home was not reported by residents [27]. Although subjective in nature and a possible source of bias, reports by residents regarding the presence of animals around the home constitute an important contribution to the epidemiology of the disease.

In a single decade, 41 human residents had ACL in 18 rural locations in the municipality of Arapongas. In 8 of the locations studied occurred human cases alone and in six locations both human and canine cases occurred. The presence of canine cases in these locations represented a 4.39-fold greater risk of human ACL in comparison to locations in which there were no cases of canine ACL. Furthermore, the relationship between the number of dogs by location and number of locations with infected dogs shows an increasing association strength increasing the risk of infection for the dog. The presence of infected dogs in the other 54 locations suggests that there is a greater possibility of dogs becoming infected in anthropogenic environments and leads to the hypothesis that canine infection precedes human infection. A possible explanation for this may be that a dog is more exposed in the area surrounding the home, where its food, water and shelter are often found. When a dog enters the forest, it does so quickly, with abrupt movements, often chasing a wild animal, which reduces the possibility of being bit by phlebotomines. Thus, the most likely time for a phlebotomine to feed on its blood is when the dog is resting in the area surrounding the residence.

The role of the dog in the transmission ACL is not fully understood. Some authors consider dogs to be secondary reservoirs of the parasite [12], [14], [37], playing some role in the cycle [15], [25], [26], [38], as a link between wild and domestic cycles [39] or as a risk factor for human disease [40]. Reithinger & Davies [41] report that current evidence on the role of domestic dogs as reservoir hosts for the domestic transmission of ACL is circumstantial. According to Dantas-Torres [42], one may expected to find dogs infected by *L.* (*V.*) *braziliensis* in endemic areas, as they are susceptible to the parasite and are often exposed to phlebotomine sandflies, meaning that dogs are not necessarily important reservoirs. One aspect to consider is that the presence of infected dogs is an indicator of the circulation of the parasite in a given environment and the risk of *L. braziliensis* infection [43].

We are aware that there are some limitations to this work. If it had been used the skin test in addition to methods that detect recent infection (parasite search and PCR in lesion, and IIF), the distribution of canine cases would probably have been different. Furthermore, as a bivariate analysis was performed cannot be ruled a possibility that a risk factor interfere in another, as in a same locality can have a forest, undergrowth and banana plants, for example.

The identification of possible risk factors for the transmission of *Leishmania* enables the implantation of measures for avoiding the disease [44]. In the locations studied, the residences, most of which do not have ceilings, were built in an unplanned, strategically poorly positioned fashion at inadequate distances from forests, streams, undergrowths and banana plants, which are propitious to the persistence of *Leishmania* [45], [46]. Other important aspects were the inappropriate discarding of household trash, which contributes to the increase in both insects and rodents. The integrated control of rodents and insects involves the combination of complementary, synergic and mutually indispensible actions [47]. Integrated environmental management could be a useful measure to avoid human contact with phlebotomines [22], [45], [48]. However, control measures for diseases transmitted by vectors have been similar throughout Brazil and do not take into account the complexity and dynamism of each location, which considerably reduces the chances of success.

### Conclusions

The results of the present study demonstrate that canine ACL infection increases the risk of human infection in the locations studied in the municipality of Arapongas (northern region of the state of Paraná, southern Brazil) and the characteristics of the area surrounding the home increase the risk of ACL in both humans and dogs. Integrated environmental management involving both the state and municipal governments as well as the population in preventive, corrective and educational actions could constitute a prophylactic measure, helping to avoid contact between humans and phlebotomine sandflies.
